# Prevalent HLA Class II Alleles in Mexico City Appear to Confer Resistance to the Development of Amebic Liver Abscess

**DOI:** 10.1371/journal.pone.0126195

**Published:** 2015-05-04

**Authors:** Eric G. Hernández, Julio Granados, Oswaldo Partida-Rodríguez, Olivia Valenzuela, Edgar Rascón, Ulises Magaña, Mónica Escamilla-Tilch, Alberto López-Reyes, Miriam Nieves-Ramírez, Enrique González, Patricia Morán, Liliana Rojas, Alicia Valadez, Alexandra Luna, Francisco J. Estrada, Carmen Maldonado, Cecilia Ximénez

**Affiliations:** 1 Laboratorio de Inmunología, Departamento de Medicina Experimental, Facultad de Medicina, UNAM, Mexico City, México; 2 División de Inmunogenética, Instituto Nacional de Ciencias Médicas y Nutrición Salvador Zubirán (INCMNSZ), SSa, Mexico City, México; 3 Departamento de Ciencias Químico-Biológicas, Universidad de Sonora, Hermosillo, Sonora, México; 4 Laboratorio de Sinovioanálisis Molecular, Instituto Nacional de Rehabilitación, SSa, Mexico City, México; 5 Laboratorio de Biología Molecular, Escuela de Medicina, Universidad Panamericana, Mexico City, México; 6 Laboratorio de Investigación en Inmunología y proteómica, Hospital Infantil de México Federico Gómez, SSa, Mexico City, México; Second Affiliated Hospital, Zhejiang University, CHINA

## Abstract

Amebiasis is an endemic disease and a public health problem throughout Mexico, although the incidence rates of amebic liver abscess (ALA) vary among the geographic regions of the country. Notably, incidence rates are high in the northwestern states (especially Sonora with a rate of 12.57/100,000 inhabitants) compared with the central region (Mexico City with a rate of 0.69/100,000 inhabitants). These data may be related to host genetic factors that are partially responsible for resistance or susceptibility. Therefore, we studied the association of the *HLA-DRB1* and *HLA-DQB1* alleles with resistance or susceptibility to ALA in two Mexican populations, one each from Mexico City and Sonora. Ninety ALA patients were clinically diagnosed by serology and sonography. Genomic DNA was extracted from peripheral blood mononuclear cells. To establish the genetic identity of both populations, 15 short tandem repeats (STRs) were analyzed with multiplexed PCR, and the allelic frequencies of HLA were studied by PCR-SSO using LUMINEX technology. The allele frequencies obtained were compared to an ethnically matched healthy control group (146 individuals). We observed that both affected populations differed genetically from the control group. We also found interesting trends in the population from Mexico City. HLA-DQB1*02 allele frequencies were higher in ALA patients compared to the control group (0.127 vs 0.047; *p*= 0.01; *pc*= NS; OR= 2.9, 95% CI= 1.09-8.3). The less frequent alleles in ALA patients were HLA-DRB1*08 (0.118 vs 0.238 in controls; *p*= 0.01; *pc*= NS; OR= 0.42, 95% CI= 0.19-0.87) and HLA-DQB1*04 (0.109 vs 0.214; *p*= 0.02; *pc*= NS; OR= 0.40, 95% CI= 0.20-0.94). The haplotype HLA-DRB1*08/-DQB1*04 also demonstrated a protective trend against the development of this disease (0.081 vs. 0.178; *p*=0.02; *pc*=NS; OR= 0.40, 95% CI= 0.16-0.93). These trends suggest that the prevalent alleles in the population of Mexico City may be associated with protection against the development of ALA.

## Introduction

Amebiasis is a significant health problem in Mexico. This endemic disease is caused by the protozoan intestinal parasite *Entamoeba histolytica* (*E*. *histolytica*) and is a major source of morbidity and mortality in the developing world. According to documents published by the World Health Organization (WHO) in 1997, amebiasis is the infection by this protozoan parasite with or without clinical manifestations [1]. Parasite cysts are transmitted through contaminated food and water, making the incidence of disease higher in areas with poor sanitation. *E*. *histolytica* is responsible for an estimated 35 to 50 million cases of symptomatic disease and approximately 100,000 deaths annually [2]. The majority of morbidity and mortality occurs in Asia, Central and South America, and Africa [3]. Although cases of amebiasis are under-reported in endemic areas, the available information ranks the disease as the second leading cause of mortality after malaria [4].

**Fig 1:**
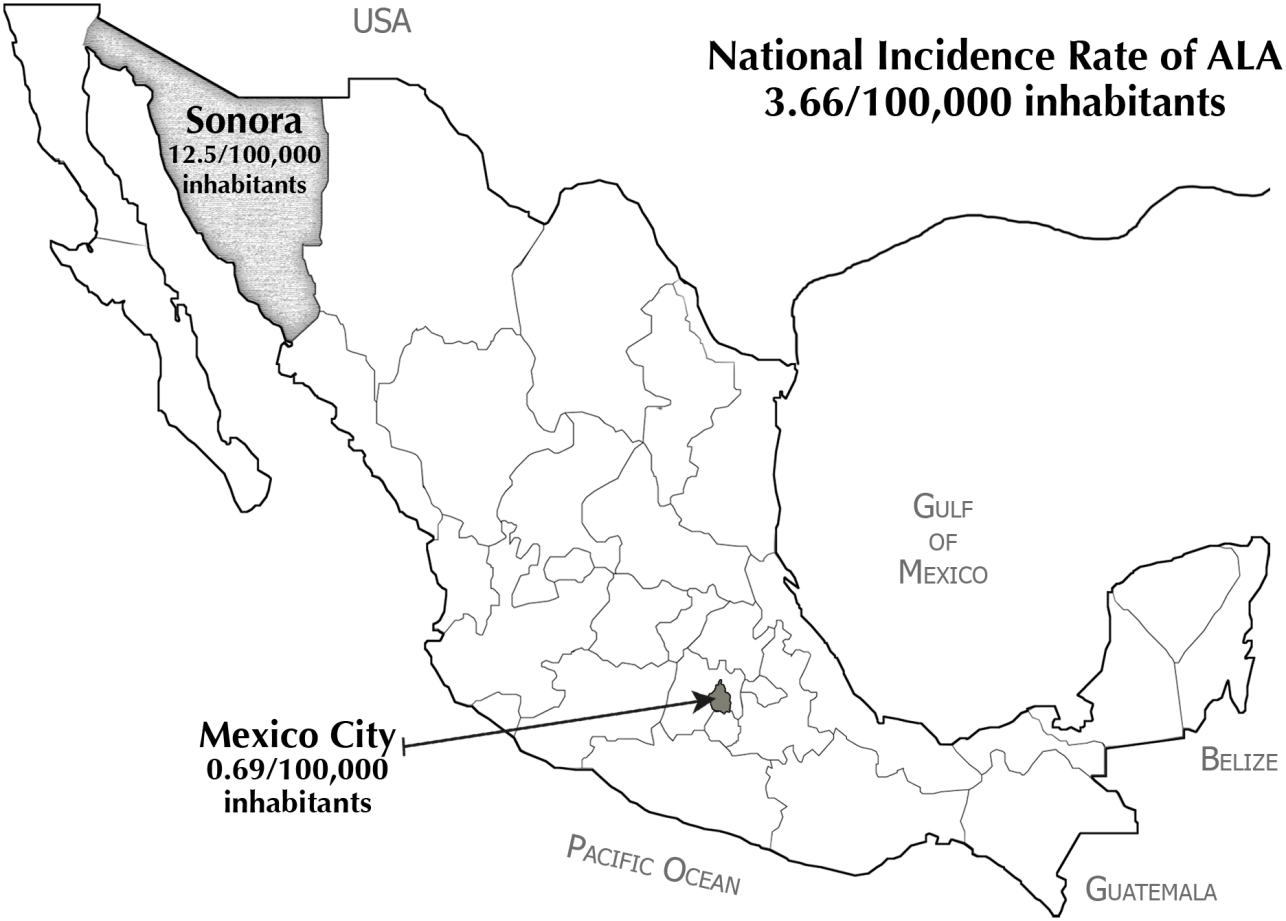
Geographical locations of Mexican population studied herein.

Amebic liver abscess (ALA) develops in less than 1% of patients infected with *E*. *histolytica* [5]. No national ALA statistics are currently kept in Mexico, and the last data available are the 2002 incidence rates. Of these, the highest rates were found in the northwestern states in the Pacific Coast of the country. One of these states is Sonora, with an incidence rate of 12.57 cases/100,000 inhabitants. This rate stands in contrast to the national average of 3.66 cases/100,000 inhabitants and the rate in Mexico City of 0.69 cases/100,000 inhabitants [6] (Fig 1). Analysis of national statistics and data on frequencies of intestinal infection obtained previously in the states of Sonora and Morelos (in the center of the country) is contradictory: Sonora displays a low frequency of intestinal infection (7%, unpublished data) but a high incidence rate of ALA [7]. On the contrary, Morelos displays a high frequency (21%) of *E*. *histolytica* and *E*. *dispar* intestinal infections [8] but a low incidence rate of ALA (3.15 cases/100,000 inhabitants).

Immune system genes are exceptionally polymorphic due to selection by a large number of rapidly varying pathogens and the need for effective elimination of these organisms to avoid the risk of self-destructive reactions [9]. These polymorphisms affect the structural domains of proteins that function in pathogen recognition and antigen presentation mechanisms. One possibility is that the diversity in the HLA genes could be partially responsible for susceptibility or resistance of human hosts to ALA. Surface molecules of *E*. *histolytica* trophozoites, such as Gal/GalNac lectin and lipopeptidophosphoglycan (LPPG), are considered virulence factors that activate antigen-presenting cells. It has been shown that macrophages and dendritic cells can internalize LPPG. The internalized LPPG is located within late endosomes, as shown by co-localization of LPPG with FITC-dextran and LAMP-1-containing organelles. In these intracellular compartments, exogenous molecules can be processed and loaded onto MHC class I, class II or CD1 molecules [10, 11]. However, we did not consider cross-presentation mechanisms in our study. Peptides from these molecules are presented through HLA class II molecules to CD4+ T cells that produce IFN-γ, initiating the primary Th1 cytokine response that activates effectors cells capable of removing the parasite [12, 13]. Previously, Arellano *et al*. reported the association of the HLA-DR3 antigen and complotype SC01 (HLA class III molecule) with ALA susceptibility in Mexican Mestizos [14,15]. Due to its biological function in the immune response, polymorphisms in genes of the HLA class II molecules (HLA-DRB1 and HLA-DQB1) are relevant candidates for studying their possible influence on the outcome of infection.

In the present work, all patients and controls were included under inclusion, exclusion and elimination criteria. Analysis of ancestry of individuals was also performed.

## Materials and Methods

### Ethics Statement

The study protocol was previously approved by the Ethical Committee of the Faculty of Medicine of the National Autonomous University of Mexico (in Spanish abbreviated as UNAM), as well as the Research and Ethics Committees of the Hospital General de México (Registration 12 CEI 09 006 13) and Hospital General del Estado de Sonora “Dr. Ernesto Ramos Bours” (Registration 13 CEI 26 030 130). Thus, the project was approved by the Ethics Committees of all participating institutions, their judgment based on the official Mexican norm NOM-012-SSA3-2007 for experimental research involving human individuals. This standard is in accordance with the Helsinki Declaration. Adult patients and controls were invited to participate in the study after being informed about the project and sampling procedures, the volunteers were asked to signed an informed consent letter to participate in this study, and in cases where the patients were children, their parents or legal mentors provided the signature of the informed written consent letter. This document is in the archives of the project, available for review by Health Ministry authorities.

### Patients and controls

The study included 90 amebic liver abscess patients clinically diagnosed by sonography and positive serology for detection of anti-amoebic antibody levels over the cutoff line defined for the Mexican population [16]. The patients included 55 from General Hospital in Mexico City and 35 from General Hospital in Hermosillo, Sonora. Control groups consisted of 84 and 62 unrelated healthy blood donors from the aforementioned hospitals, respectively, collected during the 2008–2014 period. All patients and healthy controls were Mexican Mestizos living in the same district and with the same ethnic origin and socioeconomic conditions. The reason for including this population is that Mestizos represent the majority of the Mexican population that uses the Spanish language. Spanish was used as a selection criterion because it encompasses approximately 90% of the total population in the country. In contrast, native communities are shrinking because they are being absorbed into the Mexican-Mestizo society [17]. Mexican-Mestizos are the result of admixture, principally between Amerindians and Spaniards, after the conquest of the New World. When the number of natives decreased considerably in some regions, Spaniards then brought African slaves into Mexico. Over the next 250 years, new groups, known as castes, were created [18]. The National Institute of Anthropology defines a Mexican-Mestizo as a person who was born in the country, who has a Spanish-derived last name, and has a family of Mexican ancestors back to the third generation [19].

### Inclusion and exclusion criteria

Both patients and controls were Mexican Mestizos, unrelated individuals residing in the city of study for at least two generations. In the case of ALA patients, the inclusion criteria were a clinical diagnosis supported by sonography images of hypoechoic areas and/or tomography with images of hepatic abscess and an *E*. *histolytica* seropositive result [OD greater than 0.520, (*ELISA*)] [16]. The exclusion criteria were patients with pyogenic abscesses, autoimmune diseases and other liver diseases. The control samples were obtained from healthy Mexican Mestizo blood donors, *E*. *histolytica* seronegative (lower titers of 0.520 OD, *ELISA*).

### HLA typing

A sample of 5 ml of whole blood was drawn from each individual patient and control. Genomic DNA was isolated from peripheral EDTA anti-coagulated whole blood using a modified salting-out micro technique [20]. The samples were then centrifuged to separate the leukocyte component (approximately 1 ml with 2x10^6^ cells/ml) from plasma. Erythrocytes were eliminated from the leukocyte fraction by re-suspension in a lysis buffer solution composed of sucrose, Tris-HCl 2 M, MgCl_2_ and Triton 100%. Next, the leukocytes were lysed with a second lysis buffer solution, composed of NaCl and EDTA. The DNA was separated from the proteins with SDS, NaClO_4_ and NaCl; precipitated with isopropanol at -20°C; and maintained at that temperature for 12 hours. DNA was next washed three times with 70% ethanol at -20°C. Ethanol was then decanted and the DNA was dried at room temperature and re-hydrated in sterile distilled water. When DNA was quantified for spectrophotometry, the average concentration obtained was 250 ^ng^/_μl_ in 300 μl volume, and its integrity was tested by electrophoresis in 0.8% agarose gels. Genotyping of HLA-B, HLA-DRB1 and HLA-DQB1 was performed by polymerase chain reaction sequence-specific oligonucleotide (PCR-SSO) according to the manufacturer’s specification for Lifecodes HLA-SSO typing kits. We tested for each locus using LUMINEX Technology (Gene-Probe, Inc. USA).

### STR Typing

DNA samples were also investigated for ancestry studies by testing for Short Tandem Repeat (STR) markers D8S1179, D21S11, D7S820, CSF1PO, D3S1358, TH01, D13S317, D16S539, D2S1338, D19S433, vWA, TPOX, D18S51, D5S818 and FGA loci, along with the amelogenin gene fragment. These were all co-amplified in a multiplex PCR reaction using the AmpFlSTR Identifiler Kit (Applied Biosystems, Foster City, CA) according to the manufacturer’s protocols. The amplified products, together with reference allelic ladders, were analyzed in an ABIPrism 310 Genetic Analyzer (Applied Biosystems). Capillary electrophoresis results and allele determination were analyzed using Gene Mapper software Version 3.2.

### Quality Control

Due to the slow process of collecting samples from patients and controls, DNA extractions were performed at the time of sample acquisition, thus avoiding the accumulation of samples and the possibility of cross contamination.

Sophisticated laboratory personnel processed all tests for genetic polymorphisms, and samples were reexamined if results were inconsistent.

### Statistical analysis

Allele frequency was calculated by direct counting. These frequencies were compared with matched healthy controls using χ^2^ analysis in 2x2 contingency tables, as well as with Fisher’s exact test when appropriate; *p* values that were ≤ 0.05 were considered statistically significant and corrected using the Bonferroni test. For estimation of risks, we employed odds ratios (OR) with a 95% confidence interval (95% CI). The Epi-Info statistical program was used for the estimations (version 6, Centers for Disease Control and Prevention, Atlanta, GA, USA).

## Results

A total of 90 patients were studied: 55 patients from Mexico City (43 men and 12 women) and 35 from Hermosillo, Sonora (23 men and 12 women). A group of 146 ethnically matched, healthy blood donors without symptoms or previous diagnosis of ALA, 84 from Mexico City (51 men and 33 women) and 62 from Sonora (32 men and 30 women), comprised the control groups.

### Allelic frequencies of *STRs*

To characterize our study population, the cases and controls in both locations were studied using 15 informative STRs alleles. The frequencies of the STR alleles found in this study are in agreement with previous studies in Mexican Mestizo and Mexican Native communities [21, 22, 23, 24, 25] (S1 Table). Both populations were analyzed independently (S2 and S3 Tables) and then compared to each other, revealing significant differences in five alleles (Table 1). Alleles 16 of STR *D3S1358*, 9 of STR *D13S317* and 12 of STR *TPOX* were the most common in patients and controls from Mexico City, with frequencies similar to those reported in Native American and Hispanic populations. In the patients and controls from Sonora, however, alleles 11 of STR *D13S317* and 14 of STR *D19S433* were the most common, showing similar frequencies to those of Caucasian populations [26, 27]. This result is consistent with trends of increased European genetic contributions in the northern part of Mexico [30].

**Table 1:** Different allelic frequencies of STRs between Mexico City and Sonora Populations.

STR	D3S1358		D13S317		D19S433		TPOX		
Alleles	Mexico City	Sonora	Mexico City	Sonora	Mexico City	Sonora	Mexico City	Sonora	*p* value
**9**			**0.313**	**0.196**					**0.008**
**11**			**0.168**	**0.313**					**0.001**
**12**							**0.174**	**0.063**	**0.0008**
**14**					**0.170**	**0.373**			**0.00001**
**16**	**0.341**	**0.200**							**0.001**

### Allelic frequencies of HLA class I and class II

Continuing with the characterization of our study population, HLA-B alleles were typed in 27 patient and 20 control samples from Sonora, as well as in 25 patient and 27 control samples from Mexico City. Notably, the allele HLA-B*35 is characteristic in Amerindian populations and is very common in Mexico. However, it is important to note that in Sonora, the HLA-B*14,-B*27,-B*07 and-B*44 alleles are more frequent, which suggests a European genetic component [28, 29, 30]. For individuals from Mexico City, HLA-B*35,-B*39 and-B*40 are more frequent alleles and correspond to Amerindian populations [30, 31, 32, 33, 34, 35] (Table 2).

**Table 2:** *HLA-B* frequencies in patients and controls from Sonora and Mexico City.

	Patients ALA		Controls			
	N = 27		N = 20			
*HLA-B*	n	a.f.	n	a.f.	*p*	OR (CI 95%)
B*35	9	0.166	5	0.125	0.57	1.4 (0.37–5.7)
B*14	7	0.129	3	0.075	0.39	1.8 (0.38–11.6)
B*44	7	0.129	4	0.100	0.66	1.3 (0.31–6.7)
B*07	5	0.092	2	0.050	0.43	1.9 (0.29–21.27)
B*27	5	0.092	2	0.050	0.71	1.9 (0.29–21.27)
**Mexico City**						
	Patients ALA		Controls			
	N = 26		N = 27			
***HLA-B***	**n**	**a.f.**	**n**	**a.f.**	***p***	**OR (CI 95%)**
B*35	9	0.173	10	0.185	0.87	0.9 (0.29–2.8)
B*40	8	0.153	13	0.240	0.26	0.5 (0.18–1.6)
B*39	3	0.057	11	0.203	0.02	0.2 (0.040–0.99)
B*51	6	0.115	4	0.074	0.46	1.6 (0.35–8.3)
B*15	3	0.057	3	0.055	0.96	1.0 (0.13–8.14)

**N**. Number of samples, **a.f.** Allele frequency, **OR**. Odds ratio, **CI**. Confidence interval.

With respect to frequencies of HLA class II alleles, in our population the most frequent alleles were the HLA-DRB1*04 and-DQB1*03 (Tables 3 and 4) which is consistent with the population’s ethnicity (38). In Mexico City the most prevalent alleles were the HLA-DRB1*04,-DRB1*08,-DRB1*14,-DQB1*04, considered to be markers of Amerindian populations [30, 31, 34, 36, 37] (Table 3). In contrast, in Sonora the most frequents alleles were HLA-DRB1*01,-DRB1*07 and-DQB1*02 (Table 4), all alleles commonly observed in Caucasians [30, 31, 34, 36, 38].

**Table 3:** *HLA-DRB1* and-*DQB1* allele frequency in patients and controls from Mexico City.

	Patients ALA		Controls			
	N = 55		N = 84			
*HLA-DRB1*	n	a.f.	n	a.f.	*p*	OR (CI 95%)
DRB1*04	42	0.381	47	0.279	0.07	1.5 (0.92–2.73)
DRB1*08	**13**	**0.118**	**40**	**0.238**	**0.01**	**0.42 (0.19–0.87)**
DRB1*14	7	0.063	24	0.142	0.04	0.40 (0.14–1.02)
DRB1*13	6	0.054	11	0.065	0.71	0.82 (0.24–2.52)
DRB1*15	8	0.072	8	0.047	0.38	1.5 (0.49–4.95)
DRB1*01	5	0.045	12	0.071	0.37	0.61 (0.16–1.96)
DRB1*07	7	0.063	6	0.035	0.28	1.8 (0.51–6.79)
DRB1*11	8	0.072	9	0.053	0.51	1.3 (0.44–4.18)
DRB1*16	5	0.045	6	0.035	0.68	1.2 (0.30–5.19)
DRB1*03	6	0.054	3	0.017	0.09	3.1 (0.65–19.9)
***HLA-DQB1* allele frequency**						
	Patients ALA		Controls			
	N = 55		N = 84			
***HLA-DQB1***	**n**	**a.f.**	**n**	**a.f.**	***p***	**OR (CI 95%)**
DQB1*03	67	0.609	97	0.577	0.59	1.1 (0.67–1.9)
DQB1*04	**12**	**0.109**	**36**	**0.214**	**0.02**	**0.4 (0.20–0.94)**
DQB1*06	12	0.109	14	0.083	0.47	1.3 (0.54–3.2)
DQB1*02	**14**	**0.127**	**8**	**0.047**	**0.01**	**2.9 (1.09–8.3)**
DQB1*05	5	0.045	13	0.077	0.29	0.56 (0.15–1.7)

**N**. Number of samples, **a.f.** Allele frequencies, **OR**. Odds ratio, **CI**. Confidence interval.

**Table 4:** *HLA-DRB1* and-*DQB1* allele frequency in patients and controls from Sonora.

	Patients ALA		Controls			
	N = 35		N = 62			
*HLA-DRB1*	n	a.f.	n	a.f.	*p*	OR (CI 95%)
DRB1*04	16	0.228	20	0.161	0.24	1.5 (0.68–3.41)
DRB1*01	11	0.157	12	0.096	0.21	1.7 (0.65–4.59)
DRB1*07	7	0.100	16	0.129	0.54	0.75 (0.247–2.05)
DRB1*08	8	0.114	14	0.112	0.97	1.0 (0.34–2.76)
DRB1*14	8	0.114	10	0.080	0.43	1.4 (0.47–4.37)
DRB1*11	6	0.085	10	0.080	0.90	1.0 (0.30–3.42)
DRB1*15	6	0.085	9	0.072	0.74	1.1 (0.33–3.96)
DRB1*13	5	0.071	11	0.088	0.67	0.79 (0.20–2.60)
***HLA-DQB1* allele frequency**						
	Patients ALA		Controls			
	N = 35		N = 62			
***HLA-DQB1***	**n**	**a.f.**	**n**	**a.f.**	***p***	**OR (CI 95%)**
DQB1*03	34	0.485	50	0.403	0.26	1.3 (0.74–2.62)
DQB1*02	11	0.157	32	0.258	0.10	0.53 (0.22–1.19)
DQB1*05	8	0.114	16	0.129	0.76	0.87 (0.30–2.30)
DQB1*04	8	0.114	13	0.104	0.83	1.1 (0.37–3.05)
DQB1*06	9	0.128	13	0.104	0.61	1.2 (0.44–3.39)

**N**. Number of samples, **a.f.** Allele frequencies, **OR**. Odds ratio, **CI**. Confidence interval.

### Frequencies of *HLA* class II in ALA patients and controls

In patients from Mexico City, we found that the HLA-DRB1*08 frequency was lower than in their respective controls (0.118 vs 0.238, *p* = 0.01; *pc* = NS; OR = 0.42, 95% CI = 0.19–0.87). The same result was found for HLA-DQB1*04 (0.109 vs 0.214, respectively; *p* = 0.02; *pc* = NS; OR = 0.40, 95% CI = 0.20–0.94) (Table 3). Both alleles were in linkage disequilibrium. We observed that HLA-DRB1*08/DQB1*04 was less frequent in patients, with only 9 of the 55 patients (frequency 0.081) having this haplotype, while 30 of the 84 control subjects (frequency 0.178) carried it. When we analyzed these haplotype frequencies, as expected we observed a potential protective effect against the development of ALA in this population (*p* = 0.02; *pc* = NS; OR = 0.40, 95% CI = 0.16–0.93).

We also noted that the HLA-DQB1*02 allele frequencies were higher in ALA patients compared to the control group (0.127 vs 0.047, respectively; *p* = 0.01; *pc* = NS; OR = 2.9, 95% CI = 1.09–8.3).

The distribution of alleles HLA-DRB1 and-DQB1 in the State of Sonora was very similar between ALA patients and controls (Table 4). This result prevented us from observing any important differences.

## Discussion

The relationship between humans and parasites is particularly complex. Currently, the mechanism that defines the outcome of *E*. *histolytica* infection in humans remains unknown. Is it the parasite strain’s genotype or phenotype or the host’s gender, genetic loci and environmental factors that defines the clinical form of infection? Conclusive evidence to answer this question has yet to be found. Nevertheless, the geographical differences in morbidity due to ALA in Mexico suggest that host genetic factors could be partially responsible for resistance or susceptibility to the invasive disease.

MHC molecules play an important role in the induction of immune responses, and the evolution of MHC polymorphism is often explained in terms of increased protection of hosts against pathogens. Two selective pressures that are thought to be involved are (i) selection favoring MHC heterozygous hosts and (ii) selection for rare MHC alleles by host-pathogen co-evolution [39].

The HLA alleles vary between ethnically distinct populations. Several studies have suggested that the alleles that confer resistance to certain pathogens are prevalent in areas where those pathogens cause endemic diseases. Greater resistance to infectious diseases occurs in persons that are heterozygous for specific HLA alleles because a heterozygous individual would have a broader spectrum of peptides to bind the T lymphocytes [40, 41]. These alleles also vary from one disease to another due to differences in microorganism pathogenesis [42].

Here, we were able to genetically differentiate between patients and controls in both Mexico City and Sonora using STRs, HLA-B,-DRB1 and-DQB1 alleles. It is important to emphasize that the allele frequencies in the individuals studied were consistent with those reported for the Mexican population. The different genetic backgrounds in the populations studied (Sonora and Mexico City) support the differences in the rates of morbidity. However, whether the polymorphism of HLA class II molecules serves as a risk or protection factor in the development of ALA is still unresolved. We identified a higher frequency allele, HLA-DQB1*02, in patients from Mexico City (Table 3). This allele was found to be in linkage disequilibrium with the HLA-DR3 previously reported as associated with susceptibility to ALA in pediatric patients from Mexico City [14, 15]. Interestingly, this allele is characteristic of European populations, so it probably arose in the Mexican population by admixture with European individuals. ALA patients from Mexico City exhibited a lower frequency of HLA-DRB1*08 and-DQB1*04 with respect to controls. These alleles are known to be highly frequent in Amerindians and are in linkage disequilibrium. Moreover, the HLA-DRB1*08-DQB1*04 haplotype, as expected, had a lower frequency in patients compared to controls. Unfortunately, after applying the Bonferroni’s correction to these results, differences between groups lost their statistically significant value. However, these trends suggest that the prevalent alleles in the population of Mexico City may be associated with protection against the development of ALA.

In Sonora, DRB1*01, DRB1*07, DQB1*02 and DQB1*05 were the most frequent alleles, but they did not display any association, as the distribution of these alleles in both patient and control groups was the same. This may be due to the size of the sample and/or the diversity of the geographic origins of the individuals studied. Therefore, this question requires further study using a larger sample size from Sonora. Nevertheless, we note that the HLA-DRB1*08 and-DQB1*04 alleles are present at low frequencies in the Sonoran population.

Several examples support the hypothesis that alleles that confer resistance to certain pathogens are prevalent in areas where they cause endemic diseases. These include the DRB1*13:02-DQB1*05:*01* haplotype, common in West African individuals but rare in other racial groups, is independently associated with protection from severe malaria [43]. In a cohort of 171 severe *Plasmodium falciparum* malaria patients and 101 control samples in Mumbai, the DQB1*02:*03* allele was decreased in patients relative to controls [44]. In a group of 176 Brazilian patients with Chagas disease and 448 controls, the HLADQB1*06 allele was found to afford protection against development of the disease [45]. Furthermore, in 85 seropositive and 87 seronegative individuals in Arequipa, Peru, the HLA-DRB1*14-DQB1*03:*01* haplotype was associated with resistance to infection by *Trypanosoma cruzi*, [46]. Additionally, a haplotype that protected against chronic Chagas disease, DRB1*01-B*14, was described in the Bolivian population [47].

Duggal *et al*. (2004) demonstrated associations between HLA class II alleles and protection against amebiasis in Dhaka, Bangladesh, which is an endemic country. This group found an association of the HLA class II DQB1*06:*01* allele and the DQB1*06:01/DRB1*15:*01* haplotype with protection against intestinal amebiasis. These are common Asian class II haplotypes that have been reported in other Oriental populations [48]. In this study, 129 children infected by *E*. *histolytica* and 56 uninfected individuals were typed using PCR-SSO [49]. In 2011, Duggal *et al*. reported that the Q223R polymorphism in the leptin receptor gene (outside the HLA loci) correlated with susceptibility to *E*. *histolytica* intestinal infection in Bangladeshi infants and in adult males with ALA [50].

Differences between these groups demonstrate the importance of studying the association of relevant genes with the disease in endemic countries. Additionally, the question of how HLA alleles or haplotypes are selected to promote or restrict the development of ALA in population exposed to *E*. *histolytica* parasite needs further investigation. Preliminarily, it seems that the prevalent HLA alleles in these populations are efficient at presenting antigens to CD4^+^ T cells.

Our study was limited by having a relatively small sample size. However, our results are sufficient to suggest that a protective trend is clear in the case of the DRB1*08 and DQB1*04 alleles and haplotype. These observed trends will need to be confirmed in the near future by an increased number of patients and controls from Mexico City. Moreover, this is the first molecular study of HLA class II in Mexico since the Arellano *et al*. publication 23 years ago, which used a microcytotoxicity method for detecting HLA-DR antigens in a group of pediatric patients with this disease.

Finally, this study confirms the impressive genetic diversity of Mexicans [51], and partially explains the discrepancies in clinical genetic traits for the same disease.

## Supporting Information

S1 Table Allelic frequencies of STRs in the two Mexican populations studied. (DOCX)

S2 Table Allelic frequencies of STRs from Sonora population. (DOCX)

S3 Table Allelic frequencies of STRs from the Mexico City population. (DOCX)
