# Supplementary material for: Prevalent HLA Class II Alleles in Mexico City Appear to Confer Resistance to the Development of Amebic Liver Abscess
Source: PLoS One. 2015 May 4;10(5):e0126195. doi: 10.1371/journal.pone.0126195 (PMC4418702; doi:10.1371/journal.pone.0126195)
Supplement: S1 Table — (DOCX) [file pone.0126195.s001.docx]

**S1 Table**. Allelic frequencies of STRs in the two Mexican populations studied.

| **S1 Table**. Frequencies of all STRs alleles observed in the two Mexican populations studied | | | | | | | | | | | | | | | |
| --- | --- | --- | --- | --- | --- | --- | --- | --- | --- | --- | --- | --- | --- | --- | --- |
| **Alleles** | **D8S1179** | **D21S11** | **D7S820** | **CSF1PO** | **D3S1358** | **TH01** | **D13S317** | **D16S539** | **D2S1338** | **D19S433** | **vWA** | **TPOX** | **D18S51** | **D5S818** | **FGA** |
| **6** |  |  |  |  |  | **0.275** |  |  |  |  |  |  |  |  |  |
| **7** |  |  |  |  |  | **0.367** |  |  |  |  |  |  |  |  |  |
| **8** |  |  |  |  |  |  |  |  |  |  |  | **0.539** |  |  |  |
| **9** |  |  |  |  |  |  | **0.251** |  |  |  |  |  |  |  |  |
| **10** |  |  | **0.230** | **0.286** |  |  |  | **0.210** |  |  |  |  |  |  |  |
| **11** |  |  | **0.306** | **0.286** |  |  | **0.245** | **0.241** |  |  |  | **0.252** |  | **0.446** |  |
| **12** |  |  | **0.191** | **0.312** |  |  | **0.214** | **0.300** |  |  |  |  |  | **0.296** |  |
| **13** | **0.317** |  |  |  |  |  |  |  |  | **0.177** |  |  | **0.118** |  |  |
| **14** | **0.320** |  |  |  |  |  |  |  |  | **0.279** |  |  | **0.175** |  |  |
| **15** |  |  |  |  | **0.449** |  |  |  |  |  |  |  | **0.121** |  |  |
| **16** |  |  |  |  | **0.265** |  |  |  |  |  | **0.350** |  | **0.121** |  |  |
| **17** |  |  |  |  |  |  |  |  | **0.155** |  | **0.251** |  | **0.161** |  |  |
| **18** |  |  |  |  |  |  |  |  |  |  | **0.166** |  |  |  |  |
| **19** |  |  |  |  |  |  |  |  | **0.248** |  |  |  |  |  |  |
| **20** |  |  |  |  |  |  |  |  | **0.152** |  |  |  |  |  |  |
| **21** |  |  |  |  |  |  |  |  |  |  |  |  |  |  | **0.112** |
| **22** |  |  |  |  |  |  |  |  |  |  |  |  |  |  | **0.126** |
| **23** |  |  |  |  |  |  |  |  | **0.141** |  |  |  |  |  | **0.115** |
| **24** |  |  |  |  |  |  |  |  |  |  |  |  |  |  | **0.179** |
| **25** |  |  |  |  |  |  |  |  |  |  |  |  |  |  | **0.143** |
| **26** |  |  |  |  |  |  |  |  |  |  |  |  |  |  | **0.109** |
| **29** |  | **0.197** |  |  |  |  |  |  |  |  |  |  |  |  |  |
| **30** |  | **0.313** |  |  |  |  |  |  |  |  |  |  |  |  |  |
