# Supplementary material for: Prevalent HLA Class II Alleles in Mexico City Appear to Confer Resistance to the Development of Amebic Liver Abscess
Source: PLoS One. 2015 May 4;10(5):e0126195. doi: 10.1371/journal.pone.0126195 (PMC4418702; doi:10.1371/journal.pone.0126195)
Supplement: S2 Table — (DOCX) [file pone.0126195.s002.docx]

**S2 Table**. Allelic frequencies of STRs from Sonora population.

| **S2 Table**. Allelic frequencies of STRs from Sonora population | | | | | | | | | | | | | | | |
| --- | --- | --- | --- | --- | --- | --- | --- | --- | --- | --- | --- | --- | --- | --- | --- |
| **Alleles** | **D8S1179** | **D21S11** | **D7S820** | **CSF1PO** | **D3S1358** | **TH01** | **D13S317** | **D16S539** | **D2S1338** | **D19S433** | **vWA** | **TPOX** | **D18S51** | **D5S818** | **FGA** |
| **6** |  |  |  |  |  | **0.231** |  |  |  |  |  |  |  |  |  |
| **7** |  |  |  |  |  | **0.352** |  |  |  |  |  |  |  |  |  |
| **8** |  |  |  |  |  |  |  |  |  |  |  | **0.573** |  |  |  |
| **9** |  |  |  |  |  |  | **0.196** |  |  |  |  |  |  |  |  |
| **10** |  |  | **0.200** | **0.313** |  |  | **0.090** | **0.226** |  |  |  |  |  |  |  |
| **11** |  |  | **0.300** | **0.303** |  |  | **0.313** | **0.221** |  |  |  | **0.268** |  | **0.421** |  |
| **12** |  |  | **0.215** | **0.281** |  |  | **0.234** | **0.300** |  |  |  | **0.063** |  | **0.321** |  |
| **13** | **0.326** |  |  |  |  |  |  |  |  | **0.136** |  |  | **0.122** |  |  |
| **14** | **0.305** |  |  |  |  |  |  |  |  | **0.373** |  |  | **0.154** |  |  |
| **15** |  |  |  |  | **0.447** |  |  |  |  |  |  |  | **0.117** |  |  |
| **16** |  |  |  |  | **0.200** |  |  |  |  |  | **0.313** |  | **0.111** |  |  |
| **17** |  |  |  |  |  |  |  |  | **0.191** |  | **0.228** |  | **0.159** |  |  |
| **18** |  |  |  |  |  |  |  |  |  |  | **0.159** |  |  |  |  |
| **19** |  |  |  |  |  |  |  |  | **0.223** |  |  |  |  |  |  |
| **20** |  |  |  |  |  |  |  |  | **0.164** |  |  |  |  |  |  |
| **21** |  |  |  |  |  |  |  |  |  |  |  |  |  |  | **0.115** |
| **22** |  |  |  |  |  |  |  |  |  |  |  |  |  |  | **0.131** |
| **23** |  |  |  |  |  |  |  |  | **0.101** |  |  |  |  |  | **0.136** |
| **24** |  |  |  |  |  |  |  |  |  |  |  |  |  |  | **0.173** |
| **25** |  |  |  |  |  |  |  |  |  |  |  |  |  |  | **0.115** |
| **26** |  |  |  |  |  |  |  |  |  |  |  |  |  |  | **0.084** |
| **29** |  | **0.218** |  |  |  |  |  |  |  |  |  |  |  |  |  |
| **30** |  | **0.297** |  |  |  |  |  |  |  |  |  |  |  |  |  |
