# Supplementary material for: Prevalent HLA Class II Alleles in Mexico City Appear to Confer Resistance to the Development of Amebic Liver Abscess
Source: PLoS One. 2015 May 4;10(5):e0126195. doi: 10.1371/journal.pone.0126195 (PMC4418702; doi:10.1371/journal.pone.0126195)
Supplement: S3 Table — (DOCX) [file pone.0126195.s003.docx]

**S3 Table**. Allelic frequencies of STRs from the Mexico City population.

| **S3 Table**. Allelic frequencies of STRs from the Mexico City population | | | | | | | | | | | | | | | |
| --- | --- | --- | --- | --- | --- | --- | --- | --- | --- | --- | --- | --- | --- | --- | --- |
| **Alleles** | **D8S1179** | **D21S11** | **D7S820** | **CSF1PO** | **D3S1358** | **TH01** | **D13S317** | **D16S539** | **D2S1338** | **D19S433** | **vWA** | **TPOX** | **D18S51** | **D5S818** | **FGA** |
| **6** |  |  |  |  |  | **0.325** |  |  |  |  |  |  |  |  |  |
| **7** |  |  |  |  |  | **0.385** |  |  |  |  |  |  |  |  |  |
| **8** |  |  |  |  |  |  |  |  |  |  |  | **0.500** |  |  |  |
| **9** |  |  |  |  |  |  | **0.313** |  |  |  |  |  |  |  |  |
| **10** |  |  | **0.265** | **0.256** |  |  | **0.132** | **0.192** |  |  |  |  |  |  |  |
| **11** |  |  | **0.313** | **0.268** |  |  | **0.168** | **0.265** |  |  |  | **0.234** |  | **0.475** |  |
| **12** |  |  | **0.162** | **0.347** |  |  | **0.192** | **0.301** |  |  |  | **0.174** |  | **0.268** |  |
| **13** | **0.307** |  |  |  |  |  |  |  |  | **0.225** |  |  | **0.114** |  |  |
| **14** | **0.337** |  |  |  |  |  |  |  |  | **0.170** |  |  | **0.198** |  |  |
| **15** |  |  |  |  | **0.451** |  |  |  |  |  |  |  | **0.126** |  |  |
| **16** |  |  |  |  | **0.341** |  |  |  |  |  | **0.391** |  | **0.132** |  |  |
| **17** |  |  |  |  |  |  |  |  | **0.114** |  | **0.277** |  | **0.162** |  |  |
| **18** |  |  |  |  |  |  |  |  |  |  | **0.174** |  |  |  |  |
| **19** |  |  |  |  |  |  |  |  | **0.277** |  |  |  |  |  |  |
| **20** |  |  |  |  |  |  |  |  | **0.138** |  |  |  |  |  |  |
| **21** |  |  |  |  |  |  |  |  |  |  |  |  |  |  | **0.108** |
| **22** |  |  |  |  |  |  |  |  |  |  |  |  |  |  | **0.120** |
| **23** |  |  |  |  |  |  |  |  | **0.186** |  |  |  |  |  | **0.090** |
| **24** |  |  |  |  |  |  |  |  |  |  |  |  |  |  | **0.186** |
| **25** |  |  |  |  |  |  |  |  |  |  |  |  |  |  | **0.174** |
| **26** |  |  |  |  |  |  |  |  |  |  |  |  |  |  | **0.138** |
| **29** |  | **0.174** |  |  |  |  |  |  |  |  |  |  |  |  |  |
| **30** |  | **0.331** |  |  |  |  |  |  |  |  |  |  |  |  |  |
